# A Scalable, Programmable Neural Stimulator for Enhancing Generalizability in Neural Interface Applications

**DOI:** 10.3390/bios14070323

**Published:** 2024-06-28

**Authors:** Meng Yin, Xiao Wang, Liuxindai Zhang, Guijun Shu, Zhen Wang, Shoushuang Huang, Ming Yin

**Affiliations:** 1 State Key Laboratory of Digital Medical Engineering, School of Biomedical Engineering, Hainan University, Haikou 570228, China; myin@hainanu.edu.cn (M.Y.);; 2 Key Laboratory of Biomedical Engineering of Hainan Province, One Health Institute, Hainan University, Haikou 570228, China

**Keywords:** neurostimulator, stimulation resolution, SoC, compliance voltage, neural interface

## Abstract

Each application of neurostimulators requires unique stimulation parameter specifications to achieve effective stimulation. Balancing the current magnitude with stimulation resolution, waveform, size, and channel count is challenging, leading to a loss of generalizability across broad neural interfaces. To address this, this paper proposes a highly scalable, programmable neurostimulator with a System-on-Chip (SOC) capable of 32 channels of independent stimulation. The compliance voltage reaches up to ±22.5 V. A pair of 8-bit current-mode DACs support independent waveforms for source and sink operations and feature a user-selectable dual range for low-current intraparenchymal microstimulation with a resolution of 4.31 μA/bit, as well as high current stimulation for spinal cord and DBS applications with a resolution of 48.00 μA/bit, achieving a wide stimulation range of 12.24 mA while maintaining high-resolution biological stimulation. A dedicated communication protocol enables full programmable control of stimulation waveforms, effectively improving the range of stimulation parameters. In vivo electrophysiological experiments successfully validate the functionality of the proposed stimulator. This flexible stimulator architecture aims to enhance its generality across a wide range of neural interfaces and will provide more diverse and refined stimulation strategies.

## 1. Introduction

In recent years, the rapid development of biomedicine and microelectronics has enriched our understanding of neural connectivity and neuroplasticity mechanisms. This has led to the development of more compact and functionally comprehensive neural interfaces, providing promising technologies and opportunities for addressing and improving diseases affecting the nervous system. As a typical neural interface, neural stimulators have been widely applied. For instance, deep brain stimulation (DBS) is well known for its effective control of primary tremors and Parkinson’s disease [1,2,3,4], smf it also offers some assistance in treating Alzheimer’s disease [5]. Spinal cord stimulation (SCS) is used to alleviate chronic pain caused by spinal cord injury [6,7,8] and to help patients with spinal cord injuries regain walking ability [9,10]. Moreover, electrical stimulation plays an indispensable role in epilepsy suppression [11,12], visual neuroprostheses [13], and cochlear implants for hearing assistance [14]. As the application of stimulators in various fields continues to grow, higher standards are set for their design and performance. This means that stimulators must possess higher precision-control capabilities and adaptability to diverse therapeutic needs.

Achieving ideal clinical outcomes through electrical stimulation is a challenging process, as the effectiveness of the stimulation is influenced by a variety of factors. The precise placement of electrodes, as well as the material and design of the electrodes, can affect the distribution and transmission efficiency of the current within the tissue. Tissue characteristics, including differences in resistivity, conductivity, and anatomical structures of various tissues, can also lead to variations in stimulation effects or individual patient responses to stimulation. However, the selection of stimulation parameters has a direct impact on the effectiveness of the stimulation [15,16,17,18]. By optimizing parameters such as the intensity, frequency, polarity, duration of stimulation, interphase, and interpulse delay, it is possible to significantly improve therapeutic outcomes and achieve better clinical benefits. For example, interphase and interpulse delays are essential in reducing muscle contractions and/or sensations of pain [19]. The cathodic phase of stimulation can induce action potentials, leading to muscle contraction, while the anodic phase neutralizes the charge accumulation from the cathodic phase, causing muscle relaxation. Therefore, an appropriate interphase delay can result in a good cathodic action potential without being immediately interrupted by the anode. Consequently, the stimulator’s ability to control the stimulation pulses and its own programmability is of paramount importance.

The microstimulation of the cerebral cortex tissue through electrode implantation only requires a current of 10 μA to 150 μA to recruit activity [20,21,22]. If electrode impedance can be maintained at sufficiently low levels, neural interfaces might be able to operate with low compliance voltages. However, for high impedance electrodes [23,24] (2–3 MΩ) or for applications requiring large stimulation currents such as DBS or SCS (>1 mA), high compliance voltages are indispensable. Shulyzk et al. [25] described a solution capable of high-channel-count neural recording and stimulation, but with a compliance voltage of only 2.6 V, and electrode impedances of 10 kΩ or lower are necessary to achieve a full-scale current amplitude of 250 μA. Additionally, Biphasic pulse stimulation, as opposed to monopolar stimulation, can minimize in vivo charge accumulation to the greatest extent, maintaining the charge balance and thus preventing tissue damage [26,27].

Multi-channel, high-density stimulators that support high-resolution stimulation are instrumental in facilitating focused activation within the vicinity of the target sites [28]. Such multi-channel devices enable the simultaneous stimulation of multiple neural regions, which is crucial for complex therapeutic scenarios, including pain management and neurorehabilitation. Furthermore, the utilization of high-density electrode arrays ensures that stimulation is more effectively targeted to specific neural sites rather than being broadly distributed across surrounding tissues. This focused stimulation not only enhances therapeutic outcomes but also minimizes unnecessary impacts on adjacent healthy tissues.

To address these requirements, we introduce a highly flexible stimulator with a modular parallel design that allows each of the 32 stimulation channels to be independently programmable. The internal integration of a dedicated stimulation protocol enables the configuration of all stimulation parameters, enabling the generation of arbitrary waveforms and significantly enhancing the stimulator’s programmability and versatility across a broad range of neural interface applications. Diverse clock options facilitate control over different resolutions of stimulation parameters. The stimulator supports both biphasic and monophasic pulse outputs. The high linearity characteristic ensures that the current injected into each channel is precisely controllable. To accommodate varying electrode impedances and the need for stimulation current precision, the stimulator achieves a high compliance voltage of ±22.5 V, offering two stimulation current modes: one with a resolution of 4.31 μA/bit for microcurrent stimulation and another with a resolution of 48.00 μA/bit for high-current stimulation, with full-scale current amplitudes of 1.10 mA and 12.24 mA, respectively. We are confident that this flexible design will enable a richer array of stimulation strategies and more personalized stimulation paradigms, thereby aiding in the diagnosis and treatment of neurological disorders.

The paper is organized as follows. Section 2 primarily presents the overall architecture of the stimulation chip and the implementation of key circuits, including the bandgap reference circuit, the linear current steering digital-to-analog converter (DAC), and the high-voltage output stage. It explains the configuration commands for stimulation parameters and the specifics of the dedicated stimulation protocol. In Section 3, the electrical characterization of the device is completed, with tests conducted on the device’s amplitude characteristics, frequency response, linearity, phase characteristics, and other technical specifications. Multi-channel and multi-modal stimulation outputs are also demonstrated, highlighting the device’s high programmability and flexibility. Section 4 involves in vivo animal experiments to ensure the biocompatibility and clinical applicability of the device. Section 5 provides a summary and conclusion of the entire research.

## 2. Circuit Design

### 2.1. System Architecture

**Figure 1:**
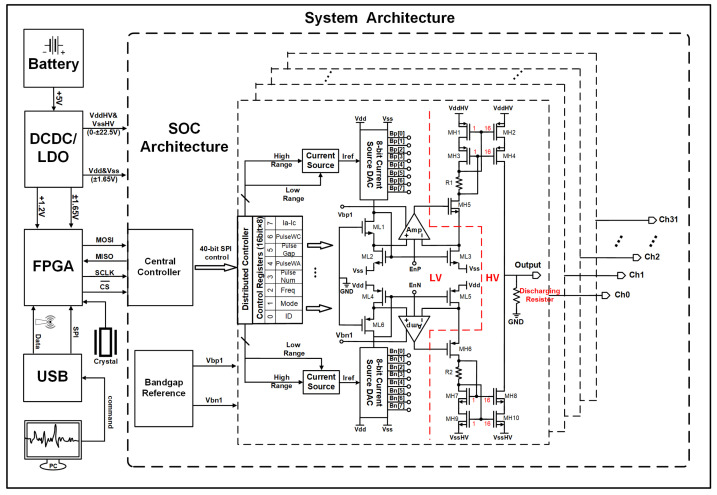
Block diagram of the biphasic stimulus system.

As depicted in Figure 1, the core of the chip comprises a central controller (CC), a bandgap reference, and 32 stimulation channels. Each channel incorporates a discrete distributed controller (DC) to process commands from the CC and generate corresponding control signals for the ends of the stimulation drive. These signals encompass various parameters such as stimulation channel selection, mode, pulse width, frequency, amplitude, and pulse delay. Current drivers produce desired stimulation pulses based on the stimulation control parameters. The 8-bit linear weight-current-steering DAC and the preceding transistors form a common-source, common-gate current mirror to generate reference currents IDACp and IDACn. The current is then mirrored to the high-voltage output stage through closed-loop regulation, and each DAC can work and be programmed independently, thus enabling single-phase or dual-phase simultaneous stimulation to achieve higher stimulation flexibility. The high-voltage output stage uses a self-biased wide swing cascade current mirror to further enhance the output capability of the stimulus current. The system is powered by a 5 V battery, and a low-dropout linear regulator can supply ±1.65 V in microcurrent stimulation mode or when using low-impedance electrodes. However, under high-current stimulation modes or high-impedance conditions, the output voltage range of the DC-DC converter can be adjusted by the sliding resistor to meet the requirements of the stimulation voltage just right. This can reduce voltage loss, thereby reducing extraneous power consumption. The range should not exceed the compliance voltage of ±22.5 V to prevent breakdown of high-voltage devices. The FPGA communicates data with the PC end and provides control commands through USB or a dedicated downloader.

### 2.2. Bandgap Reference Circuit

**Figure 2:**
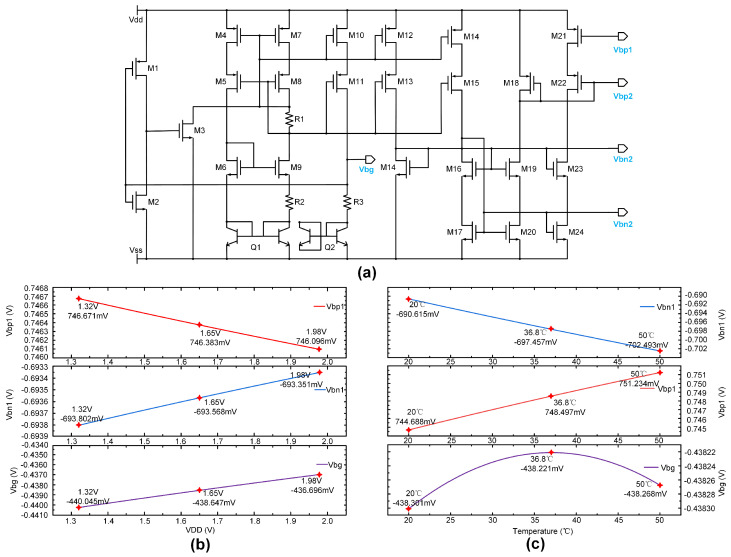
(**a**) Thermally self-biased topology bandgap reference circuit with voltage compensation. (**b**) The curve delineating the variation of the output reference voltage and bias voltage with input voltage fluctuations. (**c**) The fluctuations in the output reference voltage and bias voltage due to changes in temperature.

Inductive coupling and battery operation are two common power supply methods for implantable devices. Over extended periods of implantation, factors such as battery and coil aging, temperature fluctuations, and shifts in the position of implantation leading to a reduction in the effective coupling area of the coil can reduce their efficiency. These factors can impact the stability of the output voltage. To mitigate these effects, we have employed a bandgap reference based on a thermal voltage self-bias topology [29], employing bipolar junction transistors (Q1 and Q2) and resistors for temperature and voltage compensation. The advantage of this topology is that it can provide multiple bias voltages for use by cascaded circuits. For instance, Vbp1 and Vbn1 provide the necessary positive and negative bias voltages for the anode and cathode OTA amplifiers, respectively. The results of simulations illustrated in Figure 2b,c depict the influence of temperature and input voltage fluctuations on the output of the reference curve. With a 20% fluctuation in the power supply VDD, Vbg experienced a change of 3.35 mV, indicating that the bandgap reference has a wide input voltage range. Within a temperature range of 20 °C to 50 °C, the maximum voltage difference fluctuation of the Vbg voltage was 0.079 mV, demonstrating a high level of temperature stability in the output voltage.

### 2.3. Current-Steering DAC

**Figure 3:**
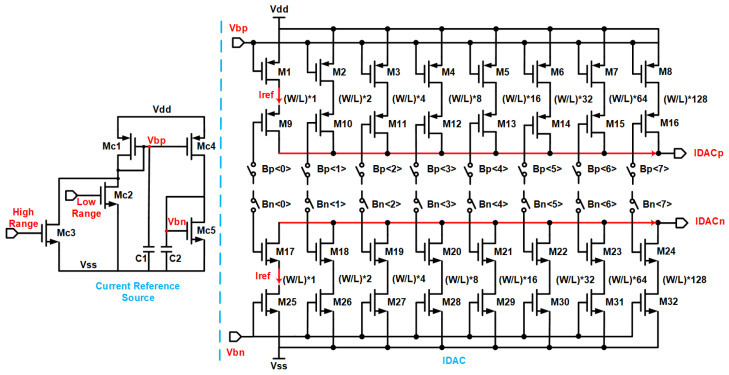
Current reference source and 8-bit binary weighted current generator.

The linearity of DACs are pivotal for ensuring the consistency and predictability of stimuli, despite the inherently high non-linearity of biological systems. A high degree of linearity in DACs can significantly reduce the effects of system non-linearities. Moreover, this aids in ensuring an active charge balance by achieving a good match between the source and sink current waveforms, hence facilitating equal charge injection and extraction. The traditional R-2R ladder DAC structure is susceptible to the impact of impedance component matching precision, although it obviates the need for numerous current sources and intricate switch-logic operations. In contrast, current steering DACs offer higher speeds, and with all current units being identical, they facilitate easier matching. The linearity loss is mitigated by incorporating dummy transistors to achieve a common centroid layout. Multi-bit DACs can offer more refined current control, which is beneficial for achieving high-resolution stimulation. High-resolution stimulation currents usually can only cover small current ranges unless the bit count of the DAC is increased. However, this entails numerous challenges, including costs and linearity issues. Figure 3 demonstrates an 8-bit current steering DAC, which provides digital control in 256 steps. The current reference source produces two different sizes of reference currents, Iref, with each current source duplicating it to generate the required current bit, yielding two resolutions of stimulation currents. All branch currents ultimately flow towards the current sinks IDACp and IDACn, which are then replicated at a high-voltage output stage and amplified. This achieves micro-stimulation currents of 4.31 μA/bit and macro-stimulation currents of 48.00 μA/bit, with full-scale currents of 1.10 mA and 12.24 mA, respectively. The switching strategy of two-level stimulation current resolution can effectively meet the dual demands of high-resolution stimulation current and a broad stimulation current output range, resolving the conflict between them.

### 2.4. High-Impedance, High-Compliance Voltage Output Stage

High current output is a critical factor in the performance of neurostimulators, particularly when faced with the challenge of significant electrode tissue impedance. This impedance can cause substantial variation in the current sink, making it imperative to adjust the operational parameters of the device accordingly. To address this issue, we have integrated 45 V high-voltage devices within the system, enabling us to offer a compliance voltage range of at least ±22.5 V. This high voltage range is critical as it allows the drain voltage of the output stage current mirror to increase in response to rises in the current sink, thereby maintaining the device’s normal function. Furthermore, to sustain the target stimulation current levels despite fluctuations in the load impedance, the stimulator is ingeniously designed with a high output impedance. This output impedance is intentionally set higher than any anticipated load impedance to guarantee a stable current output. We have also incorporated a gain-boosted current mirror, augmented by an operational amplifier, within the current sink circuit.

Furthermore, on-chip “passive discharging” resistor and off-chip AC coupling capacitors are used at the output stage to achieve passive charge balancing. This design ensures both biosafety and device safety by preventing the accumulation of charge within the system, which could otherwise lead to tissue damage or device malfunction. The “passive discharging” resistor has a high impedance of approximately 1.5 MΩ, so when connected in parallel to external electrodes, the current flowing through it is minimal. However, it can slowly discharge after the pulse ends, reducing charge accumulation before the arrival of the next stimulation cycle, and it can also provide effective protection when the circuit is unloaded.

### 2.5. Standard Digital Controller Interface

**Figure 4:**
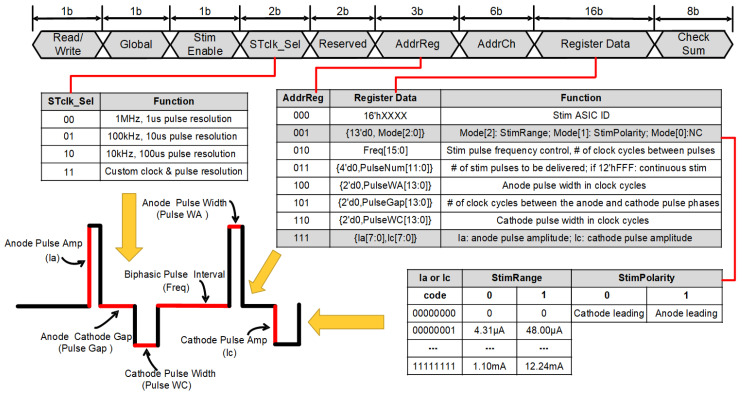
SPI stimulation parameter control commands and definitions.

To enhance the flexibility and versatility of the stimulator, we have not only optimized its performance but also integrated innovative elements and holistic designs into the stimulus control. A dedicated digital communication unit was developed to support a wide range of fully programmable stimulus waveforms, resolution and current mode switching, channel selection, enablement, clock control, and latency, among others. Such dynamic programmability facilitates the implementation of refined stimulation strategies, such as complex programmed and closed-loop stimulation, thereby increasing the device’s adaptability across diverse neural interfaces. The on-chip CC is responsible for decoding external SPI commands and then transmitting control signals to discrete DCs associated with each channel, which independently configure the stimulation parameters for their respective channels. Figure 4 illustrates a 40-Bit SPI control command, wherein “Write/Read” is utilized to execute data writing and reading operations. Once the CC receives a “Write” command, the parameters within the DC can be written and updated. The activation of the “Global” command enables synchronized stimulation across all channels; when deactivated, it allows independent stimulation for each channel. The “Stim Enable” is engaged after all register data have been sent, initiating stimulation through the channels. This functionality can be coordinated with other devices to achieve closed-loop stimulation.

By altering the LSB resolution of the clock source, each pulse width register’s interpretation is modified, which in turn directly fine-tunes the stimulation timing resolution. Through the “STclk_sel” feature, users can choose from three pre-configured clock frequencies—1 MHz, 100 kHz, and 10 kHz—that derive from an established 66 MHz external clock source. For added adaptability, the “custom clock” mode permits users to set their unique clock frequencies. “Reserved” allocates 2 bits of unused space, which hold no specific meaning but are reserved for future functional expansion. The 6-bit “AddrCh” controls individual stimulation channels, providing users with selectable options. Although there are currently only 32 stimulation channels available, the modular design allows for an easy expansion of channels. The final 8 bits are for checksum, which contains data from the preceding 32 bits, serving as a verification mechanism for the configuration commands received by the chip. Stimulation parameters are updated or maintained only when the commands are verified as a match.

**Figure 5:**
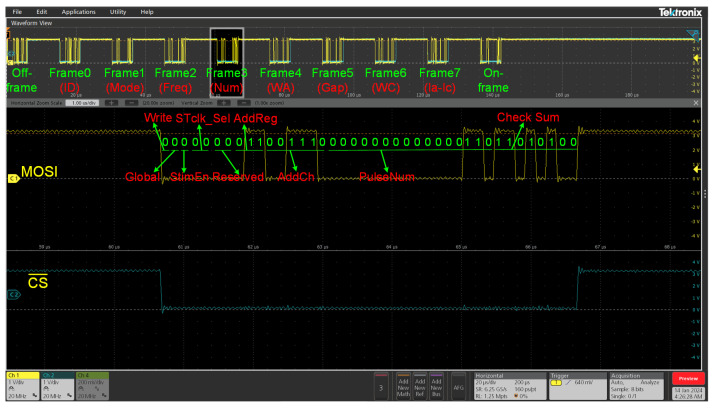
Control signal sent via MOSI when $\bar{CS}$ is low: “0 0 0 00 00 011 001110 0000000000000110 11010100”, containing the following control bit information: Read/Write = 0 (write), Global = 0, Stim Enable = 0, STclk_Sel = 00, Reserved = 00, AddrReg = 011, AddrCh = 001110, Register Data = 0000000000000110, and Checksum = 11010100. This command is used to set the clock to 1 MHz, activate Ch14, and set this channel to deliver 6 stimulation pulses.

Control parameters are encapsulated within seven 16-bit registers, covering stimulation frequency, number of pulses, biphasic pulse width, interphase delay, and current amplitude, with an additional register holding the device’s identity information. Reg000 is dedicated to storing this device identity. Reg001 defines the stimulation current mode and phase properties, where Mode[2] toggles between two distinct resolution current stimulation modes acting as the “High Range” and “Low Range”, gatekeeping current reference sources to generate the reference current. Mode[1] determines the order in which pulse phases are initiated, freely choosing between anodic leading or cathode leading prioritization. Reg010 manipulates the period between adjacent pulses. Reg011 is purposed for either dispatching a specified number of pulse cycles in sequence or for supporting continual output without interruption. Reg101 sets the interphase gap between biphasic stimulation, spacing out the cathodic and anodic phases to mitigate the effects of residual charge accumulation, ensuring that the interphase gap prevents the interruption of functional stimulation, such as muscle contraction. Reg100 and Reg110 distinctly define the duration of the anodic and cathodic pulse widths, respectively. Reg111 operates as a switch for the DAC, controlling the output stimulation current. Figure 5 outlines a 40-bit frame of data depicting the pulse quantity information.

## 3. Experiments and Measurements

**Figure 6:**
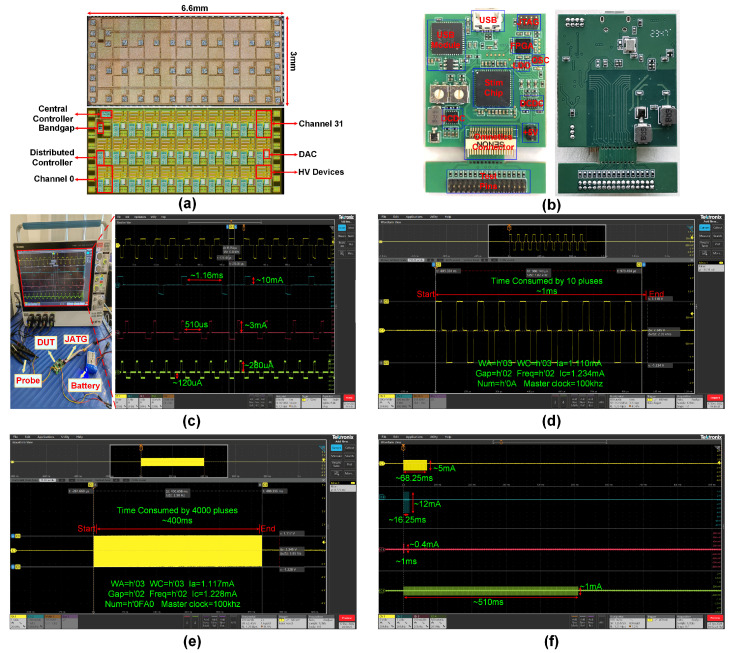
(**a**) Microphotograph (top) of 32-channel stimulator and layout (bottom). (**b**) Custom-designed stimulator test board prototype. (**c**) Independently programmable control of multi-channel stimulus current output. (**d**) PulseNum was set to h’000A to deliver 10 pulses. A complete cycle of stimulation pulses occupies 100 μs, and the continuous output of 10 stimulation pulses, as per the specified pulse count, lasts for 1 ms. (**e**) PulseNum was set to h’0FA0 to deliver 4000 pulses. A complete cycle of stimulation pulses occupies 100 μs, and the continuous output of 4000 stimulation pulses, as per the specified pulse count, lasts for approximately 400 ms. (**f**) Different duration and amplitude stimulus pulse sequences.

This SOC is designed and fabricated using the TSMC 180 nm CMOS High Voltage Mixed Signal Based Generation II BCD process. Figure 6a displays a microphotograph of the chip, showcasing its final dimensions at 3.0 mm × 6.6 mm. The chip has been indicated on the diagram, encompassing a central controller, a bandgap reference, and 32 autonomous stimulation channels. Each channel occupies an average area of 0.56 mm^2^. The chip was packaged in a standardized QFN 64-pin package and measured 9 mm × 9 mm, and a prototype of the stimulus testing board circuit, as illustrated in Figure 6b, was created. The following sections will detail the electrical measurement results of the device to demonstrate its excellent performance.

### 3.1. Multi-Channel Multi-Mode Stimulation

To cater to the diverse requirements of clinical applications, various stimulation parameters are needed depending on the specific application context. As such, the design of our system allows the independent customization of stimulation parameters for each of the 32 stimulation channels. The inherent flexibility of this design allows the configuration of the number of channels as per the user’s needs and facilitates simultaneous multi-channel stimulation output. This flexibility enables users to employ our system to investigate new neural modulation methods or to develop novel stimulation parameter paradigms for research on related neurological disorders. During the testing process, we selected four channels from the total 32 and assigned varying stimulation parameters to each. We then conducted a current through a 1 kΩ artificial electrode. The measurement and outcomes of our experiment are depicted in Figure 6c.

Ch1 to Ch3 were put into a high-current stimulation mode, while Ch4 was set to a low-current stimulation mode. For Ch1, the anodic current was set at 1.5 mA, and the cathodic current was determined to be 3 mA. Ch2 demonstrated a biphasic balanced current approximately around 10 mA. Ch3 had an anodic current of 3 mA and a cathodic current of 1.5 mA. On the contrary, Ch4 had a much lower output, with a cathodic output of merely 120 μA and an anodic output of 280 μA. Each of the channels operated with unique stimulation frequencies. Ch2 functioned with a clock period of 1.16 ms, while Ch3 was set with a pulse period of 510 μs. Despite Ch1 and Ch3 sharing the same stimulation pulse clock periods of 270 μs, analysis revealed stark differences in their stimulation waveforms.

### 3.2. Frequency Response and Phase Characteristics

**Figure 7:**
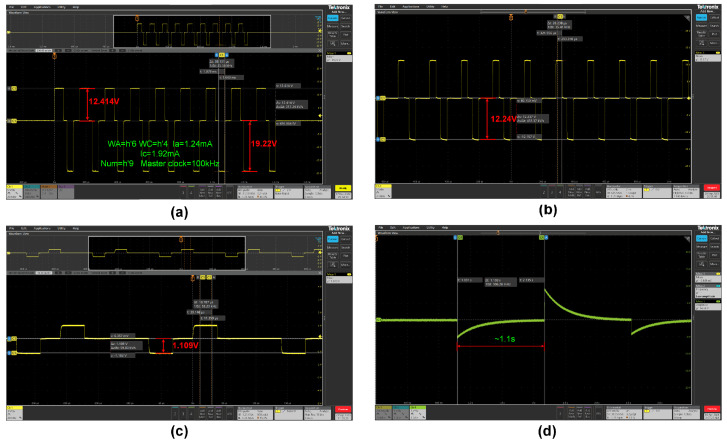
(**a**) Channel with 10 kΩ load operating in high-current stimulation mode with an anode leading configuration, 100 kHz master clock, generating a sequence of 9 stimulation pulses (PulseNum = h’9), anode leading, 60 μs anode pulse width (PulseWA = h’6), 40 μs cathode pulse width (PulseWC = h’4), 20 μs anode/cathode pulse gap (PulseGap = h’2), 40 μs biphasic pulse interval (Freq = h’4), 1.24 mA anode pulse amplitude (Ia = h’1C), and 1.92 mA cathode pulse amplitude (Ic = h’28). (**b**) High-current stimulation mode at full-scale output, load 1 kΩ, anode leading. (**c**) Microcurrent stimulation mode at full-scale output, load 1 kΩ, cathode leading. (**d**) The measured decaying exponential waveforms.

In the temporal aspect, our system allows users to customize the duration of the stimulation pulse, whereby one can set the number of individual stimulation pulses (PulseNum = h’0000 to h’0FFE) or opt for continuous, uninterrupted output (PulseNum = h’0FFF). The pulse phase can be configured to anode leading (Figure 6d and Figure 7a) or cathode leading (Ch3 in Figure 6c and Figure 7d). For a transparent demonstration of clock characteristics, the primary clock has been set to 100 kHz. Therefore, every increment of h’1 corresponds to 10 μs. We have set the anode–cathode gap to h’2 and the biphasic pulse interval (Freq = h’2). The biphasic pulse width (PulseWA and PulseWC at h’3) is determined for PulseNum set to h’A to deliver ten pulses. Considering the rigor and accuracy required for testing, the timing endpoint is recognized at the termination of the cathode of the last pulse, resulting in a time consumption of 980.194 μs. If an internal gap of each pulse is 20 μs, the total duration for ten stimulating pulses is approximately 1 ms, as represented in Figure 6d. When outputting 4000 biphasic stimulation pulses with all other parameters remaining constant (PulseNum set to h’FA0), the total duration consumed is about 400 ms, exactly 400 times that of the former case, as illustrated in Figure 6e. This showcases that the temporal characteristics are well-defined, and the measured number of pulses is also in congruence with the command sent from the controller. Utilizing this feature, the stimulator can deliver a controllable pulse sequence that includes modulation of amplitude, pulse duration, and frequency, as shown in Figure 6f.

### 3.3. Amplitude Characteristics and Linearity

To validate the device’s high compliance voltage characteristics, the stimulation power supply is provided with ±22.5 V, conducting stimulation pulses of 1.92 mA through a 10 kΩ “artificial electrode”. The measured maximum pulse amplitude of the output voltage is 19.2 V (Figure 7a). Furthermore, taking into account both the stimulation pulse widths and the charge balance, the anode pulse is configured to be 1.24 mA. To validate the linearity and amplitude characteristics of the stimulating current source output while keeping other stimulation parameters constant, the stimulation mode was set to both the high-current stimulation mode and the low-current stimulation mode. An 8-bit DAC output divided the full-scale current into 255 steps using binary coding to control the current source output. For ease of presentation, this paper uses the cathode as the reference for the maximum stimulation current. As measured in Figure 7b, in the high-current stimulation mode, the stimulating output current ranged from 0 to 12.24 mA, with a stimulation resolution of approximately 48.00 μA/bit. In the low-current stimulation mode, the full-scale current magnitude was 1.1 mA, Figure 7c, with a resolution of 4.31 μA/bit.

**Figure 8:**
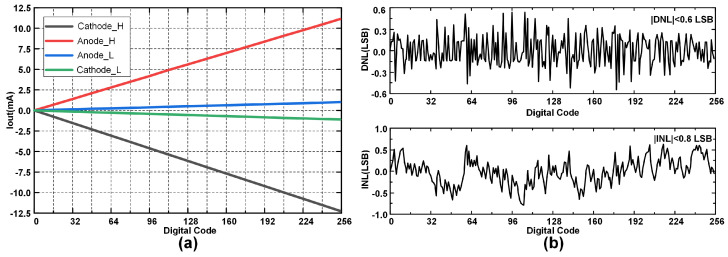
(**a**) 8-bit current source performance. (**b**) Differential nonlinearity and integral nonlinearity of the stimulator.

From the measurement results, it can be observed that the cathode output current is approximately 10% higher than the anode output current. Neglecting the influence of measurement errors, the reason for this current mismatch could be attributed to the differing mobilities of electrons and holes. The cathode and anode drivers are composed of NMOS and PMOS transistors, where the P-channel conducts holes, and the N-channel conducts electrons. Electrons have a higher mobility than holes, which means that, under the same electric field strength, the N-channel current is slightly greater than the P-channel current. However, this difference is linear and controllable, and active charge balancing can be achieved by ensuring equal charge injection and extraction using good matching between the source and sink current waveforms. Moreover, the stimulator is capable of realizing a current exponential decay waveform, as illustrated in Figure 7d. This attenuation of the current is achieved through an on-chip “passive discharging” resistor in conjunction with an external 2 MΩ electrode acting as a “shunt”. The interplay between these two components forms the basis for the current’s decay profile, optimizing it for specific stimulation protocols. Figure 8a shows the relationship between current and binary digital input, and overall, the current source has high linearity and monotonicity under both stimulus modes. The absolute values of differential nonlinearity (DNL) and integral nonlinearity (INL) are less than 0.6 LSB and 0.8 LSB, respectively, and the measurement results are shown in Figure 8b.

## 4. Electrophysiological Experiment

**Figure 9:**
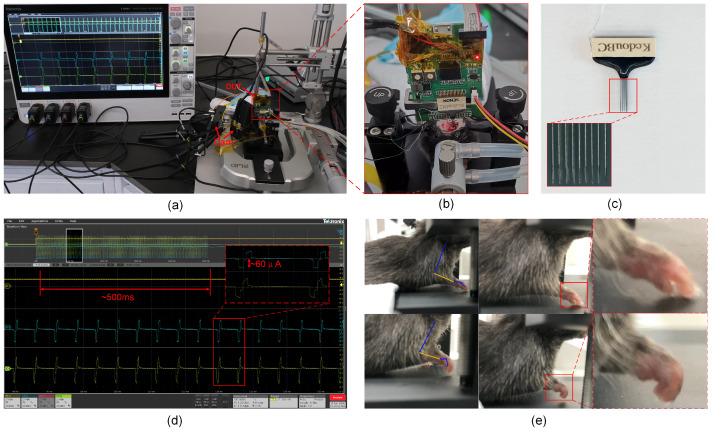
(**a**) Experimental setup for in vivo testing. (**b**) Details of device implantation. (**c**) Stimulation electrodes and micrographs. (**d**) Stimulation pulse train lasting approximately 500 ms and details of the stimulation pulse waveform. (**e**) Experimental mouse in resting state (top) and stimulation-induced contraction of the forelimb at the elbow (bottom).

We customized a microneedle array, as depicted in Figure 9c, featuring an electrode impedance of approximately 100 kΩ. This array is assembled from tungsten wires with conical tips, designed for penetration into brain tissues, spinal cords, and similar structures. An outer layer of biocompatible insulation covers the electrode wires, with an interspatial distance of 200 μm between them. The dimensions of this array are sufficient to encompass the entire M1 brain region of a mouse. This electrode array is compatible with a 32-channel Omnetics biological interface, facilitating board-to-board reliable connections with stimulators and allowing for repeated use. During the experiment, anesthesia was induced in mice using a mixture of 2% to 2.5% isoflurane gas with oxygen. The mice were then secured within a stereotaxic apparatus, positioned prone on the apparatus’s fixation platform with forelimbs exposed for craniotomy. During surgery, the skin was incised, connective tissues were removed, and a section of the skull over the M1 motor cortex was drilled to expose the underlying area. Electrode reference wires were wrapped and fixed around cranial screws, and electrodes, guided by a holder, were inserted into the cortical tissue at appropriate depths. During the stimulation period, anesthesia was maintained by controlling the gas anesthesia apparatus to achieve a concentration of 1.0–1.5% anesthesia gas, ensuring light anesthesia. The anesthesia level was monitored throughout the entire experiment by observing respiratory rate, body temperature, and reflex responses to gentle pressure on the feet and tail [30].

The in vivo experiments aimed to use the long-duration pulse stimulation (ICMS) parameter strategies involved in [31,32] to evoke forelimb movements and validate the functionality of the electrical stimulator. Specifically, biphasic stimulation pulses at a frequency of 333 Hz were employed, initiated by a cathodal phase followed by an anodal phase, with each phase lasting 200 μs. This setup facilitated the generation of pulse trains approximately 500 ms in duration through the controlled number of pulses. To swiftly detect movement, stimulation currents of 60 μA, 80 μA, and 100 μA were sequentially employed at the same stimulation site. If no movement was detected at 100 μA, the site was deemed non-responsive [33] (since the typical movement threshold current is approximately 50 μA), necessitating a change in stimulation site. Figure 9d illustrates the 500 ms pulse train and its corresponding individual stimulation pulse waveform details. The results showed that, despite utilizing identical stimulation parameters, recorded stimulation waveforms exhibited variability across different channels. This variability can likely be attributed to differences in electrode impedance and tissue impedance at different stimulation sites. Moreover, utilizing high-definition cameras to capture forelimb movements in mice, the stimulation paradigm successfully induced movements characterized by elbow joint contraction and displacement. Some sites exhibited transient muscle twitches. The upper image in Figure 9e records the forelimb in a stationary state, while the lower image shows the results of the elbow forelimb contraction induced by stimulation. Based on these experimental outcomes, the functionality of the proposed stimulator was successfully verified.

## 5. Conclusions and Future Work

In this work, we presented a highly programmable neural electrical stimulator, and proprietary serial communication protocol and stimulation driving circuit architecture significantly enhance the flexibility of the stimulator, providing a standardized communication interface. Thirty-two channels can be configured with independent stimulation waveforms. It supports optional pulse amplitude resolution selection within a current range of 0–12.24 mA, which is suitable for low-current microstimulation with a resolution of 4.31 μA/bit, as well as high-current stimulation with a resolution of 48 μA/bit. The device achieves high compliance voltages of ±22.5 V and compact size using a 180 nm process. Its functionality was demonstrated through custom PCB circuit test boards and animal experiments, with excellent performance greatly enhancing its versatility across a broad range of neural interface applications. Table 1 shows a comparison with some existing advanced stimulation devices. By comparison, it can be seen that the stimulator we propose has certain competitive advantages in terms of the number of channels, stimulation current and resolution, compliance voltage, and programmability.

In future work, the stimulator will implement more channels and higher stimulation resolution and provide more complex pattern stimulation paradigms and a rich array of stimulation strategies. Thanks to its modular design, we believe achieving these enhancements will not be difficult. At the same time, we will also design more flexible neural stimulation devices, fully considering their size and weight adaptability for small subjects. Additionally, the device will be expanded into a wireless system for neural scientific research on free, unconstrained behavior. It will also integrate neural recording capabilities for stimulation monitoring or to implement closed-loop neural modulation. These advancements will promote the development of neuroscience and be beneficial for clinical applications.

## Figures and Tables

**Table 1:** Comparison Of stimulation ASICs.

Specifications	Rozgic [34]	Yen [35]	M.R [36]	Haoran [37]	Feyerick [38]	This Work
Technology (nm)	180 HV	250	130	180 HV	65	180 HV
Supply Voltage (V)	1.8	2.5/5	3.3	1	11	±1.65
Current (mA)	0–5.1 (7b)	0–5 (6b)	0–1.35 (8b)	0–12.75 (8b)	0–0.16 (7b)	0–1.10/0–12.24 ^a^
Compliance Voltage (V)	9	10	NA	40	10.4	±22.5
Channels	8	4	16	16/4	4	32
Die Size (mm^2^)	16.287	0.88	0.176	30.25	0.2256	19.8
Charge Balance	Yes	Yes	No	Yes	Yes	Yes ^b^
Stim Type	Biphasic	Biphasic	Biphasic	Mono/Biphasic	Biphasic	Mono/Biphasic
Pulse Number	NA ^c^	NA	NA	NA	NA	0–4094/ Uninterrupted
Biphasic interval (ms)	NA	NA	NA	NA	NA	0.002–6553.5 ^d^
Pulse width (ms)	NA	YES	NA	YES	0.4	0.002–1638.3
Interphase gap (ms)	NA	NA	NA	NA	NA	0.002–1638.3

^a^ Two ranges, both with 8-bit resolution. ^b^ Passive charge balance achieved by using on-chip “passive discharging” resistor or active charge balance achieved by achieving a good match between the source and sink current waveforms. ^c^ Not Available. ^d^ Achieving a wider range or high-resolution pulse width and delay control by altering the master clock frequency.

## Data Availability

The data is presented within this article.

## References

[1] Patel B., Chiu S., Wong J.K., Patterson A., Deeb W., Burns M., Zeilman P., Wagle-Shukla A., Almeida L., Okun M.S. (2021). Deep brain stimulation programming strategies: Segmented leads, independent current sources, and future technology. Expert Rev. Med. Devices.

[2] Kuhn J., Hardenacke K., Lenartz D., Gruendler T., Ullsperger M., Bartsch C., Mai J., Zilles K., Bauer A., Matusch A. (2015). Deep brain stimulation of the nucleus basalis of Meynert in Alzheimer’s dementia. Mol. Psychiatry.

[3] Eitan R., Fontaine D., Benoît M., Giordana C., Darmon N., Israel Z., Linesky E., Arkadir D., Ben-Naim S., Iserlles M. (2018). One year double blind study of high vs low frequency subcallosal cingulate stimulation for depression. J. Psychiatr. Res..

[4] Lozano A.M., Lipsman N., Bergman H., Brown P., Chabardes S., Chang J.W., Matthews K., McIntyre C.C., Schlaepfer T.E., Schulder M. (2019). Deep brain stimulation: Current challenges and future directions. Nat. Rev. Neurol..

[5] Luo Y., Sun Y., Tian X., Zheng X., Wang X., Li W., Wu X., Shu B., Hou W. (2021). Deep brain stimulation for Alzheimer’s disease: Stimulation parameters and potential mechanisms of action. Front. Aging Neurosci..

[6] Sdrulla A.D., Guan Y., Raja S.N. (2018). Spinal cord stimulation: Clinical efficacy and potential mechanisms. Pain Pract..

[7] Mekhail N., Levy R.M., Deer T.R., Kapural L., Li S., Amirdelfan K., Hunter C.W., Rosen S.M., Costandi S.J., Falowski S.M. (2020). Long-term safety and efficacy of closed-loop spinal cord stimulation to treat chronic back and leg pain (Evoke): A double-blind, randomised, controlled trial. Lancet Neurol..

[8] Caylor J., Reddy R., Yin S., Cui C., Huang M., Huang C., Rao R., Baker D.G., Simmons A., Souza D. (2019). Spinal cord stimulation in chronic pain: Evidence and theory for mechanisms of action. Bioelectron. Med..

[9] Lorach H., Galvez A., Spagnolo V., Martel F., Karakas S., Intering N., Vat M., Faivre O., Harte C., Komi S. (2023). Walking naturally after spinal cord injury using a brain–spine interface. Nature.

[10] Collinger J.L., Vinjamuri R., Degenhart A.D., Weber D.J., Sudre G.P., Boninger M.L., Tyler-Kabara E.C., Wang W. (2014). Motor-related brain activity during action observation: A neural substrate for electrocorticographic brain-computer interfaces after spinal cord injury. Front. Integr. Neurosci..

[11] Cheng C.H., Tsai P.Y., Yang T.Y., Cheng W.H., Yen T.Y., Luo Z., Qian X.H., Chen Z.X., Lin T.H., Chen W.H. (2018). A fully integrated 16-channel closed-loop neural-prosthetic CMOS SoC with wireless power and bidirectional data telemetry for real-time efficient human epileptic seizure control. IEEE J. Solid-State Circuits.

[12] Valentin A., Ughratdar I., Cheserem B., Morris R., Selway R., Alarcon G. (2015). Epilepsia partialis continua responsive to neocortical electrical stimulation. Epilepsia.

[13] Chen K., Yang Z., Hoang L., Weiland J., Humayun M., Liu W. (2010). An integrated 256-channel epiretinal prosthesis. IEEE J. Solid-State Circuits.

[14] Jiang C., Singhal S., Landry T., Roberts I.V., de Rijk S.R., Brochier T., Goehring T., Tam Y.C., Carlyon R.P., Malliaras G.G. (2021). An instrumented cochlea model for the evaluation of cochlear implant electrical stimulus spread. IEEE Trans. Biomed. Eng..

[15] Simon M.V., Nuwer M.R., Szelényi A. (2022). Electroencephalography, electrocorticography, and cortical stimulation techniques. Handb. Clin. Neurol..

[16] Eggers T., Kilgore J., Green D., Vrabec T., Kilgore K., Bhadra N. (2021). Combining direct current and kilohertz frequency alternating current to mitigate onset activity during electrical nerve block. J. Neural Eng..

[17] Simpson H.D., Schulze-Bonhage A., Cascino G.D., Fisher R.S., Jobst B.C., Sperling M.R., Lundstrom B.N. (2022). Practical considerations in epilepsy neurostimulation. Epilepsia.

[18] Abdollahifard S., Farrokhi A., Mosalamiaghili S., Assadian K., Yousefi O., Razmkon A. (2023). Constant current or constant voltage deep brain stimulation: Short answers to a long story. Acta Neurol. Belg..

[19] Cvetkoska A., Maček-Lebar A., Trdina P., Miklavčič D., Reberšek M. (2022). Muscle contractions and pain sensation accompanying high-frequency electroporation pulses. Sci. Rep..

[20] Provenza N.R., Matteson E.R., Allawala A.B., Barrios-Anderson A., Sheth S.A., Viswanathan A., McIngvale E., Storch E.A., Frank M.J., McLaughlin N.C. (2019). The case for adaptive neuromodulation to treat severe intractable mental disorders. Front. Neurosci..

[21] Flesher S.N., Collinger J.L., Foldes S.T., Weiss J.M., Downey J.E., Tyler-Kabara E.C., Bensmaia S.J., Schwartz A.B., Boninger M.L., Gaunt R.A. (2016). Intracortical microstimulation of human somatosensory cortex. Sci. Transl. Med..

[22] London B.M., Jordan L.R., Jackson C.R., Miller L.E. (2008). Electrical stimulation of the proprioceptive cortex (area 3a) used to instruct a behaving monkey. IEEE Trans. Neural Syst. Rehabil. Eng..

[23] Cogan S.F. (2008). Neural stimulation and recording electrodes. Annu. Rev. Biomed. Eng..

[24] Suner S., Fellows M.R., Vargas-Irwin C., Nakata G.K., Donoghue J.P. (2005). Reliability of signals from a chronically implanted, silicon-based electrode array in non-human primate primary motor cortex. IEEE Trans. Neural Syst. Rehabil. Eng..

[25] Shulyzki R., Abdelhalim K., Bagheri A., Salam M.T., Florez C.M., Velazquez J.L.P., Carlen P.L., Genov R. (2014). 320-channel active probe for high-resolution neuromonitoring and responsive neurostimulation. IEEE Trans. Biomed. Circuits Syst..

[26] Luo Z., Ker M.D. (2016). A high-voltage-tolerant and precise charge-balanced neuro-stimulator in low voltage CMOS process. IEEE Trans. Biomed. Circuits Syst..

[27] Nath B., Peng S.Y., Lo Z.J., Pai Y.H., Yeh Y.T., Chang H.H., Lu Y.C., Huang S.H., Chang F.C. (2023). A biphasic current-mode stimulator integrated circuit with a novel residual charge compensation mechanism. Integration.

[28] Noorsal E., Sooksood K., Xu H., Hornig R., Becker J., Ortmanns M. (2011). A neural stimulator frontend with high-voltage compliance and programmable pulse shape for epiretinal implants. IEEE J. Solid-State Circuits.

[29] Ghovanloo M., Najafi K. (2004). A compact large voltage-compliance high output-impedance programmable current source for implantable microstimulators. IEEE Trans. Biomed. Eng..

[30] Brown A.R., Teskey G.C. (2014). Motor cortex is functionally organized as a set of spatially distinct representations for complex movements. J. Neurosci..

[31] Ramnani N. (2006). The primate cortico-cerebellar system: Anatomy and function. Nat. Rev. Neurosci..

[32] Bonazzi L., Viaro R., Lodi E., Canto R., Bonifazzi C., Franchi G. (2013). Complex movement topography and extrinsic space representation in the rat forelimb motor cortex as defined by long-duration intracortical microstimulation. J. Neurosci..

[33] Viaro R., Bonazzi L., Maggiolini E., Franchi G. (2017). Cerebellar modulation of cortically evoked complex movements in rats. Cereb. Cortex.

[34] Rozgić D., Hokhikyan V., Jiang W., Akita I., Basir-Kazeruni S., Chandrakumar H., Marković D. (2018). A 0.338 cm^3^, artifact-free, 64-contact neuromodulation platform for simultaneous stimulation and sensing. IEEE Trans. Biomed. Circuits Syst..

[35] Yen T.Y., Ker M.D. (2020). Design of dual-mode stimulus chip with built-in high voltage generator for biomedical applications. IEEE Trans. Biomed. Circuits Syst..

[36] Pazhouhandeh M.R., Kassiri H., Shoukry A., Weisspapir I., Carlen P.L., Genov R. (2020). Opamp-less sub-μW/channel Δ-modulated neural-ADC with super-GΩ input impedance. IEEE J. Solid-State Circuits.

[37] Pu H., Malekzadeh-Arasteh O., Danesh A.R., Nenadic Z., Do A.H., Heydari P. (2021). A CMOS dual-mode brain-computer interface chipset with 2-mV precision time-based charge balancing and stimulation-side artifact suppression. IEEE J. Solid-State Circuits.

[38] Feyerick M., Dehaene W. (2023). Dense, 11 V-tolerant, Balanced Stimulator IC with Digital Time-domain Calibration for <100 nA Error. IEEE Trans. Biomed. Circuits Syst..

